# Clinicopathological features, survival outcomes, and appropriate surgical approaches for stage I acinar and papillary predominant lung adenocarcinoma

**DOI:** 10.1002/cam4.3012

**Published:** 2020-03-24

**Authors:** Di Lu, Jianjun Yang, Xiguang Liu, Siyang Feng, Xiaoying Dong, Xiaoshun Shi, Jianxue Zhai, Shijie Mai, Jianjun Jiang, Zhizhi Wang, Hua Wu, Kaican Cai

**Affiliations:** ^1^ Department of Thoracic Surgery Nanfang Hospital Southern Medical University Guangzhou China

**Keywords:** acinar, lung adenocarcinoma, papillary, surgical procedures, survival

## Abstract

**Background:**

Whether prognosis differs between lung acinar predominant adenocarcinoma (ACN) and papillary predominant adenocarcinoma (PAP) patients remains controversial. Furthermore, the appropriate surgical plan for each subtype is undetermined.

**Methods:**

Data of stage I ACN or PAP patients from 2004 to 2015 were retrospectively reviewed by SEER*Stat 8.3.5. The primary outcome was overall survival (OS) and lung cancer specific survival (LCSS).

**Results:**

1531 patients (PAP, 484; ACN, 1047) were included. ACN patients had better OS (*P* = .001) and LCSS (*P* = .003) than PAP patients. Among stage I ACN patients, lobectomy with mediastinal lymph node dissection (Lob) (*P* = .001) or segmentectomy (Seg) (*P* = .003) provided a better OS than wedge resection (Wed). And ACN patients who received Lob had a equivalent LCSS, compared to those who received Seg (*P* = .895). For patients with PAP in stage I, those who received Lob tended to have a better prognosis than that received Seg (HR of OS, 0.605, 95% CI: 0.263‐1.393; HR of LCSS, 0.541, 95% CI: 0.194‐1.504) or Wed (HR of OS, 0.735, 95% CI: 0.481‐1.123; HR of LCSS, 0.688, 95% CI: 0.402‐1.180).

**Conclusions:**

Among patients with lung adenocarcinoma in stage I, those with ACN have a better OS and LCSS than that with PAP. For patients with stage I ACN, Seg and Lob, rather than Wed, seem to be an equivalent treatment choice; however, Seg is the prior option because it could preserve more lung function than Lob. For patients with PAP, Lob tends to be a better choice than Wed and Seg, although the prognostic difference between them is nonsignificant.

## Graphical abstract

This SEER based study which we aimed to evaluate the prognosis difference of acinar predominant carcinoma (ACN) and papillary predominant adenocarcinoma (PAP) patients after lobectomy with mediastinal lymph node dissection and explored the best surgical treatment for each type. Our data suggested that ACN have a better OS and LCSS than that with PAP. For patients with stage I ACN, Seg and Lob, rather than Wed, seem to be an equivalent treatment choice, and for patients with PAP, Lob tends to be a better choice than Wed and Seg, although the prognostic difference between them is nonsignificant.

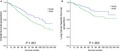

## 1 INTRODUCTION

Lung cancer is the most commonly diagnosed cancer and the leading cause of cancer death.^1^ Nonsmall cell lung cancer (NSCLC) accounts for approximately 85% of all lung cancer histological types, and up to 50% of NSCLCs are adenocarcinoma.^2^ With the successful use of computerized tomography screening for early detection of lung cancer, an increasing number of early‐stage NSCLC cases were reported, most of which were adenocarcinoma.^3^

For patients with early‐stage NSCLC, lobectomy with mediastinal lymph node dissection (Lob) has been proposed as the standard surgical procedure.^4^, ^5^ However, for some specific groups of patients with early‐stage NSCLC, a few studies showed that limited resection (LR), including segmentectomy (Seg) and wedge resection (Wed), could achieve equivalent survival compared to lob.^6^, ^7^, ^8^, ^9^ Thus, in addition to TNM stage and surgical approaches, some other factors, such as pathologic subtypes, may also affect patients’ postoperative survival. According to the classification of World Health Organization,^10^ invasive lung adenocarcinoma can be divided into several subtypes, lepidic, acinar, papillary, micropapillary, solid, fetal adenocarcinoma, enteric adenocarcinoma, and etc Overall, the following prognostic associations were reported: patients with solid and micropapillary adenocarcinoma have the worst prognosis, those with nonmucinous lepidic adenocarcinoma have the best outcome, while those with acinar predominant adenocarcinoma (ACN) and papillary predominant adenocarcinoma (PAP) have intermediate survival.^11^, ^12^, ^13^, ^14^ The difference in prognosis between patients with ACN and those with PAP, however, remains ambiguous.^12^, ^15^, ^16^

Taken together, it seems safe for patients with early‐stage lung solid and micropapillary adenocarcinoma to receive lob^17^, ^18^ and acceptable for patients with early‐stage nonmucinous lepidic adenocarcinoma to receive LR.^19^ However, to the best of our knowledge, no studies have found the best surgical approach for patients with early‐stage ACN and PAP.

To address these issues of great interest, the Surveillance, Epidemiology, and End Results (SEER) public database was employed, which is a national population‐based database and provides both large cohort size and long‐term follow‐up.

Thus, the aim of this study was to evaluate the postoperative differences in prognosis between NSCLC patients with ACN and PAP and to determine the best surgical approaches based on the SEER public database.

## 2 MATERIALS AND METHODS

### 2.1 Patient selection and study parameters

**Figure 1:**
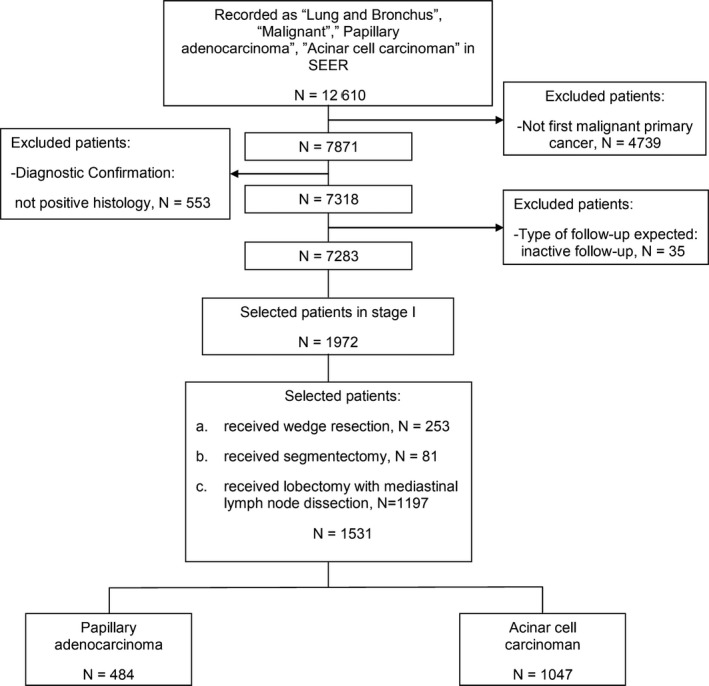
Flow diagram of the patient selection process. SEER: Surveillance, Epidemiology, and End Results public database

Patients in this study were identified from the SEER public database. SEER*Stat 8.3.5 was used to extract data of patients with ACN and PAP from 2004 to 2015 (Figure 1). Patients were primarily identified using the term “Lung and Bronchus” and “papillary adenocarcinoma”(8260/3) and “acinar cell carcinoma”(8550/3). The variable, “Sequence number”, was used to identify the patients with a single primary tumor. Patients whose diagnosis was not histologically confirmed were excluded. In addition, patients with inactive follow‐up were excluded. Patients in stage II, III, IV, or unknown stage were excluded. The variable, “RX Summ‐‐Surg Prim Site,” was used to identify patients who underwent Wed (21), Seg (22), and Lob (33). The following characteristics were extracted from the dataset: age, gender, race, tumor size, TNM stage (AJCC ‐ 6th Edition), grade of differentiation, and treatment history of radiotherapy and chemotherapy.

### 2.2 Statistical Analysis

The primary outcome of this study was overall survival (OS) and lung cancer specific survival (LCSS). Follow‐up duration was calculated from 2004 to 2015. All data were analyzed using the SPSS software package, version 23.0 (IBM, SPSS Statistics). Pearson's chi‐squared test was used to analyze differences between the groups. Propensity score matching (PSM) was used to create two groups of ACN and PAP patients with similar profiles, paired on a 1:1 ratio. The Kaplan‐Meier method was used to estimate OS and LCSS. The log‐rank test was performed to make comparisons of survival curves between subgroups. Statistical significance was defined as *P* < .05. And multivariate Cox regression was used to control the confounding factors between the three groups of surgical approaches. The Cox proportional hazards regression model was used to estimate hazard ratio(HR) of OS and LCSS for prognostic factors, including age, gender, race, T stage, grade of differentiation, tumor size, surgical types, and treatment history of radiotherapy and chemotherapy. Variables with a *P*‐value that is less than .1 or of clinical significance were included in the multivariate model.

## 3 RESULTS

### 3.1 Patients’ characteristics

A total of 1531 patients with lung adenocarcinoma were selected from the SEER database (Figure 1). As shown in Table 1, among 1531 patients, there were 1047 ACN patients and 484 PAP patients. The difference was significant in gender (*P* = .002), tumor size (*P* < .001), and grade of differentiation (*P* < .001) between ACN and PAP group, while there was no significant difference in age at diagnosis, race, T stage, and treatment history of radiotherapy chemotherapy and surgery. In order to control these confounding factors between the two groups, PSM was employed. After a 1:1 PSM, there was no statistically significant difference in age at diagnosis, race, gender, T stage, tumor size, grade of differentiation, and treatment history of radiotherapy, chemotherapy, or surgery.

**Table 1:** Comparison of the clinicopathological characteristics of patients with ACN and PAP before and after PSM

Characteristics	ACN (N = 1047)	PAP (N = 484)	*P*‐value	ACN (N = 484)	PAP (N = 484)	*P*‐value
	Before PSM			After PSM		
Age			.428			.344
<60	215 (20.5)	108 (22.3)		96 (19.8)	108 (22.3)	
≥60	832 (79.5)	376 (77.7)		388 (80.2)	376 (77.7)	
Gender			**.002**			.651
Male	387 (37.0)	219 (45.2)		212 (43.8)	219 (45.2)	
Female	660 (63.0)	265 (54.8)		272 (56.2)	265 (54.8)	
Race			.628			.329
White	853 (81.5)	385 (79.5)		403 (83.3)	385 (79.5)	
Black	78 (7.4)	42 (8.7)		35 (7.2)	42 (8.7)	
Others	116 (11.1)	57 (11.8)		46 (9.5)	57 (11.8)	
T stage			.199			.148
T1	644 (61.5)	281 (58.1)		303 (62.6)	281 (58.1)	
T2	403 (38.5)	203 (41.9)		181 (37.4)	203 (41.9)	
Grade			**<.001**			.335
Well or moderately differentiated	825 (78.8)	419 (86.6)		429 (88.6)	419 (86.6)	
Poorly or undifferentiated	145 (13.8)	30 (6.2)		31 (6.4)	30 (6.2)	
Unknown	77 (7.4)	35 (7.2)		24 (5.0)	35 (7.2)	
Tumor size			**<.001**			.152
≤3	854 (81.6)	339 (70.0)		359 (74.2)	339 (70.0)	
>3	193 (18.4)	145 (30.0)		125 (25.8)	145 (30.0)	
Radiotherapy			.471			.374
No	1034 (98.8)	480 (99.2)		483 (99.8)	480 (99.2)	
Yes	13 (1.2)	4 (0.8)		1 (0.2)	4 (0.8)	
Chemotherapy			.071			.071
No/Unknown	977 (93.3)	439 (90.7)		454 (93.8)	439 (90.7)	
Yes	70 (6.7)	45 (9.3)		30 (6.2)	45 (9.3)	
Surgery			.159			.599
Wed	181 (17.3)	72 (14.9)		63 (13.0)	72 (14.9)	
Seg	61 (5.8)	20 (4.1)		17 (3.5)	20 (4.1)	
Lob	805 (76.9)	392 (81.0)		404 (83.5)	392 (81.0)	

Abbreviations: CAN, acinar predominant adenocarcinoma; Lob, lobectomy with mediastinal lymph node dissection; PAP, papillary predominant adenocarcinoma; PSM, propensity score matching; Seg, segmentectomy; Wed, wedge resection.

The *P*‐value of the difference of differentiation grade between ACN and PAP group is .0000607 and the *P*‐value of the difference of tumor size between ACN and PAP group is .00000043 (in bold).

### 3.2 Survival analysis

#### 3.2.1 Differences in prognosis between patients with ACN and PAP

**Figure 2:**
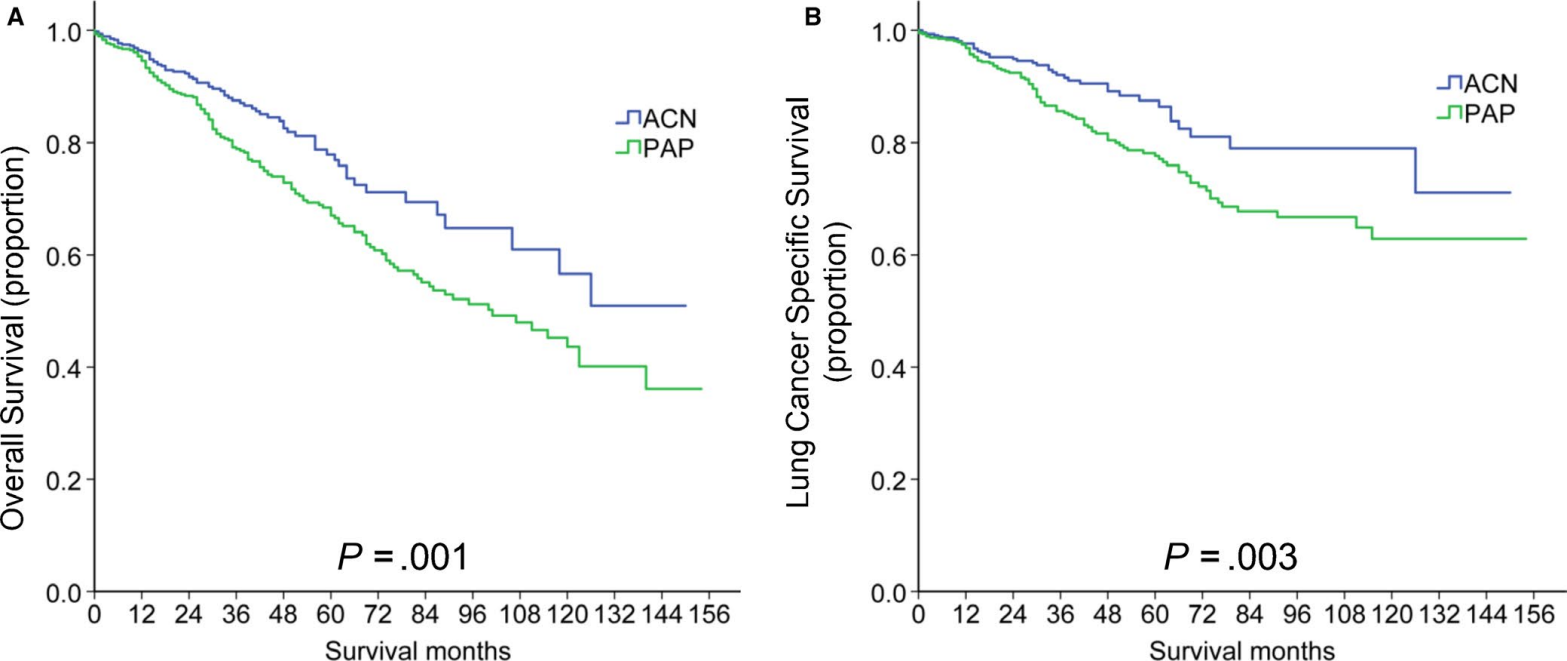
Kaplan‐Meier survival analysis for overall survival (A) and lung cancer specific survival (B) of patients with ACN and PAP in stage I. ACN, acinar predominant adenocarcinoma; PAP, papillary predominant adenocarcinoma

After PSM, the 968 patients, including 484 PAP patients and 484 ACN patients, were divided into two groups according to the pathological types. As shown in Figure 2A, patients with ACN had a more favorable OS than PAP patients (*P* = .001), with a 3‐year OS rate and 5‐year OS rate of 87.5% (95% CI: 83.97%‐91.03%) and 77.9% (95% CI: 72.22%‐83.58%), while those with PAP had a 3‐year OS rate and 5‐year OS rate of 78.9% (95% CI: 74.98%‐82.82%) and 67.0% (95% CI: 61.90%‐72.10%). PAP patients had a median OS of 101 months, while those with ACN did not reach their median OS (mean of OS: 109.532 ± 4.517 months).

We then compared the differences in LCSS between patients with ACN and PAP. As shown in Figure 2B, ACN patients had a significantly better LCSS than those with PAP (*P* = .003). Both ACN patients and PAP patients did not reach their median LCSS, with a mean of LCSS of 126.08 ± 3.95 months and 115.83 ± 3.42 months respectively. Patients with ACN and those with PAP had a 3‐year LCSS rate of 92.1% (95% CI: 89.16% ‐ 95.04%) and 85.6% (95% CI: 82.07% ‐ 89.13%), respectively, and had a 5‐year LCSS rate of 87.5% (95% CI: 83.19% ‐ 91.81%) and 77.6% (95% CI: 72.90% ‐ 82.30%) respectively.

### 3.3 Differences in prognosis between patients with ACN in stage I who received Wed, Seg, and Lob

**Figure 3:**
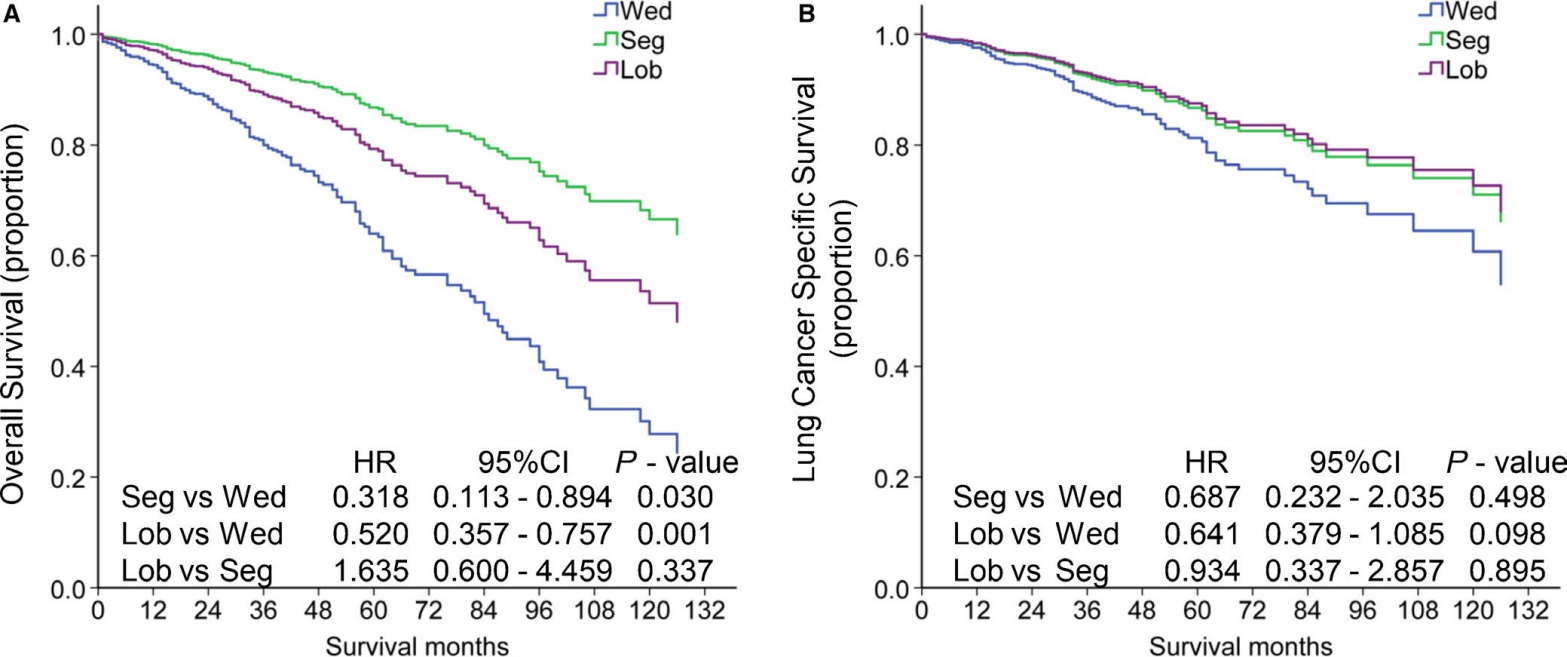
Kaplan‐Meier survival analysis for overall survival (A) and lung cancer specific survival (B) of patients with ACN in stage I according to the surgery type. ACN, acinar predominant adenocarcinoma; Wed, wedge resection; Seg, segmentectomy; Lob, lobectomy with mediastinal lymph node dissection

Among 1047 patients with ACN, 181, 61, 805 patients received Wed, Seg, and Lob respectively. In order to investigate the differences in prognosis between patients with ACN in Stage I who received Wed, Seg, and Lob, these patients were divided into three groups according to the procedure that they received. Multivariate Cox regression was used to control the confounding factors (Table S1), including age, gender, race, T stage, grade of differentiation, tumor size, the histories of whether receive radiotherapy, or chemotherapy, in the three groups. As shown in Figure 3A, patients who received Seg or Lob had a significantly better OS, compared to those underwent Wed (Wed vs Seg, *P* = .003; Wed vs Lob, *P* = .001, Seg vs Lob, *P* = .337). The HR of patients who underwent Seg and Lob was 0.318 (95% CI: 0.113‐0.894) and 0.520 (95% CI: 0.357‐0.757), compared to those who received Wed. Furthermore, the HR of patients who underwent Lob was 1.635 (95% CI: 0.600‐4.459), compared to patients received Seg.

The differences in prognosis of LCSS between the three groups were also assessed after controlling the confounding factors by multivariate Cox regression (Table S2). As shown in figured 3B, patients who received Lob (HR, 0.934, 95% CI: 0.337‐2.857) had a similar LCSS compared to those underwent Seg (*P* = .895). While compared to those who received Wed, patients who underwent Seg and Lob had a HR of 0.687 (95% CI: 0.232‐2.035) and 0.641 (95% CI: 0.379‐1.085) respectively.

### 3.4 Differences in Prognosis between patients with PAP in stage I who received wed, seg, and lob

**Figure 4:**
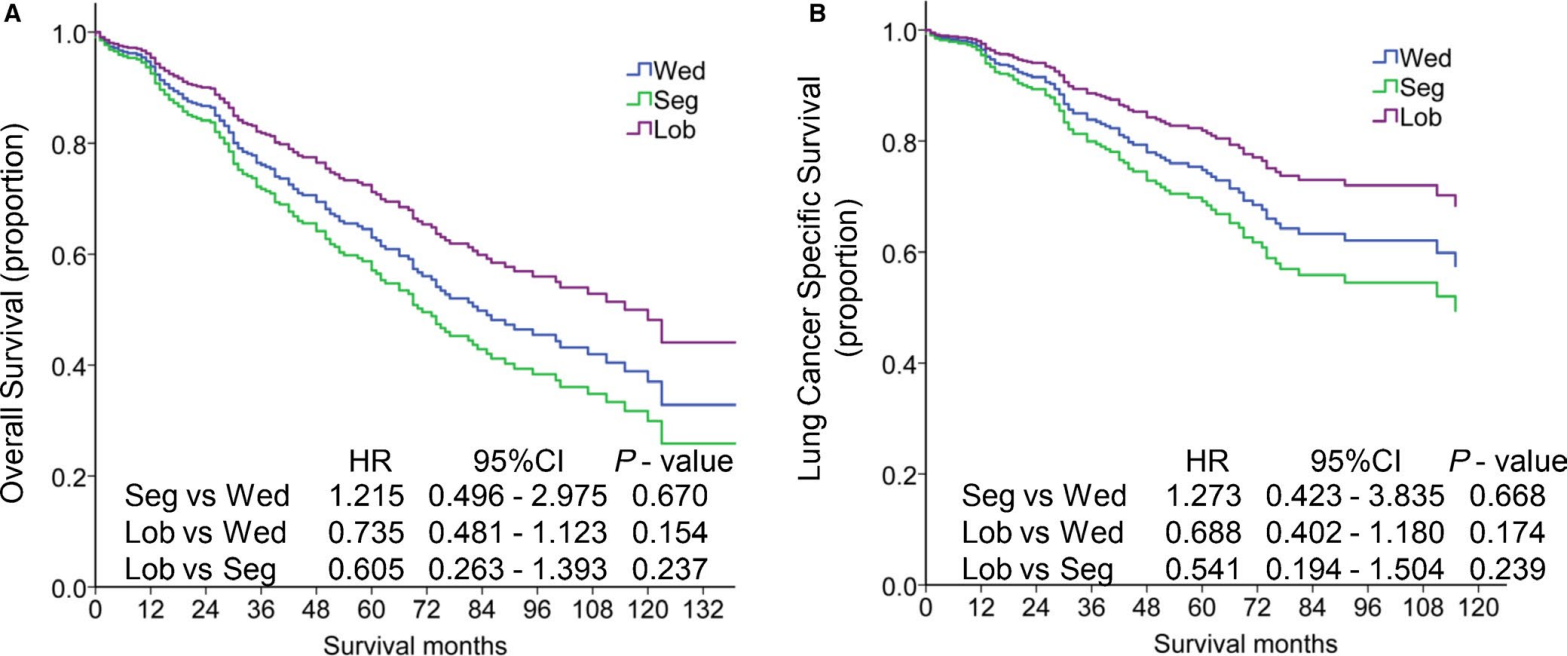
Kaplan‐Meier survival analysis for overall survival (A) and lung cancer specific survival (B) of patients with PAP in stage I according to the surgery type. Lob, lobectomy with mediastinal lymph node dissection; PAP, papillary predominant adenocarcinoma; Wed, wedge resection; Seg, segmentectomy

Among 484 patients with PAP, 72, 20, 392 patients received Wed, Seg, and Lob respectively. In order to evaluate the prognostic differences between patients in Stage I who received Wed, Seg, and Lob, PAP patients were divided into three groups according to the procedure that they received. Multivariate Cox regression was used to control the confounding factors (Table S3), including age, gender, race, T stage, grade of differentiation, tumor size, the histories of whether receive radiotherapy or chemotherapy, in the three groups. As shown in Figure 4A, the HR of patients who underwent Seg and Lob was 1.215 (95% CI: 0.496‐2.975) and 0.735 (95% CI: 0.481‐1.123), compared to those who received Wed. Furthermore, the HR of patients who underwent Lob was 0.605 (95% CI: 0.263‐1.393), compared to patients who received Seg.

The differences in prognosis of LCSS between the three groups were also assessed. As shown in Figure 4B, after controlling the confounding factors by multivariate Cox regression (Table S4), patients who underwent Seg and Lob had a HR of 1.273 (95% CI: 0.423‐3.835) and 0.688 (95% CI: 0.402‐1.180), respectively, compared to those received Wed. Further more, the HR of patients who underwent Lob was 0.541 (95% CI: 0.194‐1.504), compared to patients who received Seg.

### 3.5 Prognostic factors of OS in patients with ACN and PAP

To investigate the factors affecting the prognosis of patients with ACN and PAP, clinicopathologic characteristics were assessed for OS and LCSS by univariate and multivariate Cox regression analyses.

For patients with ACN (N = 1047), the univariate analysis (Table S1) indicated that elder age (*P* = .002), male gender (*P* = .028), T2 (*P* < .001), lower grade of differentiation (*P* < .001), larger tumor size (*P* < .001), treatment history of receiving radiotherapy (*P* < .001), and receiving Wed (Wed vs Seg, *P* = .008, Wed vs Lob, *P* = .001) were related to worse OS. In multivariate analysis (Table S1), elder age (*P* = .025), T2 (*P* < .001), poor or undifferentiated differentiation (*P* = .01), and receiving Wed (Wed vs Seg, *P* = .003, Wed vs Lob, *P* = .001) were associated with shorter OS. The effect of these clinicopathologic characteristics on LCSS of patients with ACN were further assessed. According to the results from univariate analysis (Table S2), T2 (*P* < .001), poor or undifferentiated differentiation (*P* < .009), larger tumor size (*P* < .001), receiving radiotherapy (*P* = .001) was associated with worse LCSS. In the multivariate analysis (Table S2), ACN patients in T2 stage (*P* = .001) and received radiotherapy (*P* = .012) had a shorter LCSS.

For patients with PAP (N = 484), as shown in table S3, the univariate analysis indicated that elder age (*P* = .005), T2 stage (*P* = .001), larger tumor size (*P* < .001), treatment history of receiving chemotherapy (*P* = .014) were associated with worse OS. From the results of multivariate analysis (Table S3), elder age (*P* = .011 was associated with shorter OS. We the focus on the effect of clinicopathologic characteristics on LCSS of patients with PAP. In univariate analysis (Table S4), T2 stage (*P* < .001), lower grade of differentiation (*P* = .01), larger tumor size (*P* < .001) and receiving chemotherapy were related to worse LCSS. The results of multivariate analysis (Table S4) indicated that patients with unknown grade of differentiation and received chemotherapy had a shorter LCSS.

## 4 DISCUSSION

In this study, we found that among patients with lung adenocarcinoma in stage I, those with ACN had a significantly better OS and LCSS than patients with PAP. Some studies have evaluated the prognostic difference between patients with ACN and PAP and showed a trend similar to that of our present study, although the reported difference was not statistically significant. Yoshizawa et al reported 5‐year survival rates of 81.2% and 74.4% for patients with ACN and those with PAP respectively.^13^ The trend of Yoshizawa's study is in line with our study, although their OS curves of the two subtypes were not clearly separated. It might be caused by the small sample size of Yoshizawa's study (ACN, N = 61; PAP, N = 179). Similarly, in a Japanese cohort,^20^ patients with ACN (N = 59) had a 5‐year disease‐free survival rate of 83.7%, while this rate in patients with PAP (N = 16) was 75.0%. However, some studies suggested that patients with ACN and PAP had a similar prognosis. In an American study,^11^ patients with PAP (N = 143) and ACN (N = 232) had 5‐year disease‐free survival rates of 83% and 84% respectively (5‐year OS rate was not mentioned). In an Australian cohort,^15^ the OS curves of the two subtypes were not clearly separated. In Yanagawa's study, PAP patients (N = 40) and ACN patients (N = 40) had 5‐year disease‐free survival rates of 85.4% and 89.7% respectively.^21^ It should be noted that all these studies lack a large sample size. To the best of our knowledge, this study is the largest cohort that analyzed the prognostic difference between ACN and PAP. Thus, it seems that patients with ACN had a better OS than those with PAP, and a larger sample trial is needed to confirm this conclusion.

The appropriate surgical approach for PAP and ACN has not been fully investigated yet. According to our study, for patients with ACN in stage I, those received Seg or Lob had similar prognoses, which are better than that of patients who underwent Wed. Similar trend was observed in the analysis of LCSS, though the difference in prognosis was not statistically significant. This may be explained by the observation that Seg and Lob have a similar effect on stage I lung adenocarcinoma.^22^, ^23^ These results suggest that for patients with ACN, Seg seemed to be an equivalent treatment choice compared to Lob. However, Seg could preserve more lung function and provide a better quality than Lob. For patients with stage I PAP, those received Lob tended to have a better OS and LCSS than those received Seg and Wed, though the difference was not statistically significant. The nonsignificant tests for the OS and LCSS may be explained by lack of power because of the small number of endpoint events and lack of a large sample size. These results suggest that for patients with PAP in stage I, Lob tends to remain to be the better option of surgical approach for them, unless they are in bad general condition. The effect of Wed, Seg, and lob on early‐stage NSCLC has been widely evaluated.^6^, ^7^, ^24^ However, it should be noted that these studies ignored the effect of histological subtypes of invasive lung adenocarcinoma on OS and LCSS. To the best of our knowledge, the present study is the first research to investigate the appropriate surgical approaches for patients with ACN and PAP. More cohorts with large‐scale samples are needed to validate this conclusion.

Our present study has some limitations. Although the entire cohort of our study was large, the patients were not uniformly distributed in every subgroup. This may cause a lack of power of the analysis in the subgroup. Furthermore, in addition to the clinical and pathologic characteristics investigated in our study, other factors such as smoking status, EGFR mutation status, KRAS mutation status may also affect the survival of patients with lung adenocarcinoma.^25^, ^26^ Unfortunately, these data are not captured in SEER database.

In conclusion, ACN patients might have a better OS and LCSS than those with PAP. For patients with stage I ACN, Seg and Lob, rather than Wed, seem to be an equivalent treatment choice; however, Seg is the prior option because it could preserve more lung function and provide a better quality for them than Lob. For patients with PAP, Wed, Seg, and Lob show similar prognosis for those in stage I, and Lob tends to be a better choice although the difference between them is nonsignificant.

## CONFLICT OF INTEREST

The authors declare that the research was conducted in the absence of any commercial or financial relationships that could be construed as a potential conflict of interest.

## AUTHOR CONTRIBUTIONS

DL, JY, and XL performed data analyses. DL, JY, JZ, and JJ wrote the manuscript. SF, XD, SM, and XS contributed in data collection. HW revised the manuscript. ZW processed the figures. KC designed the study.

## DATA SHARING STATEMENT

The datasets used and/or analyzed during the current study are available from the corresponding author on reasonable request.

## Supporting information

Table S1‐S4

## Data Availability

The data of this study are available from the corresponding author upon reasonable request.
