# Supplementary material for: Clinicopathological features, survival outcomes, and appropriate surgical approaches for stage I acinar and papillary predominant lung adenocarcinoma
Source: Cancer Med. 2020 Mar 24;9(10):3455–62. doi: 10.1002/cam4.3012 (PMC7221422; doi:10.1002/cam4.3012)
Supplement: Supplementary file 1 — Table S1‐S4 [file CAM4-9-3455-s001.doc]

Supplemental table 1. Univariate and multivariate analysis of overall survival in patients with ACN (N = 1047).

| **Characteristics** | **Univariate analysis** | | | **Multivariable analysis** | | |
| --- | --- | --- | --- | --- | --- | --- |
|  | **HR** | **95%CI** | **p-value** | **HR** | **95%CI** | **p-value** |
| **Age** |  |  |  |  |  |  |
| ＜60 | 1 |  |  | 1 |  |  |
| ≥60 | 2.014 | 1.299-3.122 | **0.002** | 1.673 | 1.068-2.620 | **0.025** |
| **Gender** |  |  |  |  |  |  |
| Male | 1 |  |  | 1 |  |  |
| Female | 0.721 | 0.539-0.965 | **0.028** | 0.776 | 0.578-1.041 | 0.091 |
| **Race** |  |  | 0.171 |  |  | 0.268 |
| White | 1 |  |  | 1 |  |  |
| Black | 0.895 | 0.485-1.649 | 0.721 | 0.813 | 0.438-1.509 | 0.511 |
| Others | 0.544 | 0.287-1.032 | 0.062 | 0.606 | 0.318-1.155 | 0.128 |
| **T stage** |  |  |  |  |  |  |
| T1 | 1 |  |  | 1 |  |  |
| T2 | 2.588 | 1.927-3.478 | **＜0.001** | 2.203 | 1.547-3.136 | **＜0.001** |
| **Grade** |  |  | **0.001** |  |  | **0.032** |
| Well or moderatelydifferentiated | 1 |  |  | 1 |  |  |
| Poorly or undifferentiated | 1.89 | 1.351-2.643 | **＜0.001** | 1.573 | 1.113-2.223 | **0.01** |
| Unknown | 1.431 | 0.807-2.538 | **0.22** | 1.318 | 0.741-2.343 | 0.347 |
| **Tumor size** |  |  |  |  |  |  |
| ≤3 | 1 |  |  | 1 |  |  |
| ＞3 | 2.114 | 1.544-2.896 | **＜0.001** | 1.212 | 0.823-1.785 | 0.33 |
| **Radiotherapy** |  |  |  |  |  |  |
| No | 1 |  |  | 1 |  |  |
| Yes | 3.273 | 1.447-7.402 | **0.004** | 2.278 | 0.970-5.350 | 0.059 |
| **Chemotherapy** |  |  |  |  |  |  |
| No/Unknown | 1 |  |  | 1 |  |  |
| Yes | 1.228 | 0.753-2.003 | **0.41** | 0.851 | 0.509-1.424 | 0.539 |
| **Surgery** |  |  | **0.001** |  |  | **0.001** |
| Wed | 1 |  |  | 1 |  |  |
| Seg | 0.251 | 0.090-0.699 | **0.008** | 0.318 | 0.113-0.894 | **0.03** |
| Lob^†^ | 0.558 | 0.393-0.791 | **0.001** | 0.52 | 0.357-0.757 | **0.001** |
| Lob^‡^ | 2.225 | 0.823-6.014 | **0.115** | 1.635 | 0.600-4.459 | 0.337 |

Abbreviation: ACN: acinar predominant adenocarcinoma; HR, hazard ratio; CI, confidence interval; Wed: wedge resection; Seg: segmentectomy; Lob: lobectomy with mediastinal lymph node dissection; †: Lob was compared to Wed; ‡: Lob was compared to Seg.

Supplemental table 2. Univariate and multivariate analysis of lung cancer specific survival in patients with ACN (N = 1047).

| **Characteristics** | **Univariate analysis** | | | **Multivariate analysis** | | |
| --- | --- | --- | --- | --- | --- | --- |
|  | **HR** | **95% CI** | ***p*-value** | **HR** | **95% CI** | ***p*-value** |
| **Age** |  |  |  |  |  |  |
| ＜60 | 1 |  |  | 1 |  |  |
| ≥60 | 1.624 | 0.952-2.768 | 0.075 | 1.301 | 0.753-2.247 | 0.345 |
| **Gender** |  |  |  |  |  |  |
| Male | 1 |  |  | 1 |  |  |
| Female | 0.689 | 0.472-1.007 | 0.054 | 0.739 | 0.504-1.085 | 0.123 |
| **Race** |  |  | 0.447 |  |  | 0.381 |
| White | 1 |  |  | 1 |  |  |
| Black | 0.548 | 0.202-1.493 | 0.24 | 0.493 | 0.180-1.354 | 0.17 |
| Others | 0.825 | 0.416-1.636 | 0.582 | 0.901 | 0.451-1.800 | 0.767 |
| **T stage** |  |  | **＜0.001** |  |  |  |
| T1 | 1 |  |  | 1 |  |  |
| T2 | 3.052 | 2.059-4.523 | **＜0.001** | 2.602 | 1.631-4.151 | **＜0.001** |
| **Grade** |  |  | **0.031** |  |  | 0.24 |
| Well or moderately differentiated | 1 |  |  | 1 |  |  |
| Poorly or undifferentiated | 1.808 | 1.161-2.815 | **0.009** | 1.472 | 0.934-2.321 | 0.096 |
| Unknown | 1.278 | 0.588-2.778 | 0.536 | 1.233 | 0.565-2.692 | 0.599 |
| **Tumor size** |  |  |  |  |  |  |
| ≤3 | 1 |  |  | 1 |  |  |
| ＞3 | 2.395 | 1.604-3.575 | **＜0.001** | 1.299 | 0.796-2.118 | 0.295 |
| **Radiotherapy** |  |  |  |  |  |  |
| No | 1 |  |  | 1 |  |  |
| Yes | 4.605 | 1.871-11.336 | **0.001** | 3.404 | 1.303-8.894 | **0.012** |
| **Chemotherapy** |  |  |  |  |  |  |
| No/Unknown | 1 |  |  | 1 |  |  |
| Yes | 1.319 | 0.705-2.470 | 0.386 | 0.813 | 0.420-1.574 | 0.54 |
| **Surgery** |  |  | 0.268 |  |  | 0.253 |
| Wed | 1 |  |  | 1 |  |  |
| Seg | 0.521 | 0.179-1.518 | 0.232 | 0.687 | 0.232-2.035 | 0.498 |
| Lob^†^ | 0.7 | 0.432-1.133 | 0.147 | 0.641 | 0.379-1.085 | 0.098 |
| Lob^‡^ | 1.344 | 0.493-3.668 | 0.564 | 0.934 | 0.337-2.857 | 0.895 |

Abbreviation: ACN: acinar predominant adenocarcinoma; HR, hazard ratio; CI, cnfidence interval; Wed: wedge resection; Seg: segmentectomy; Lob: lobectomy with mediastinal lymph node dissection; †: Lob was compared to Wed; ‡: Lob was compared to Seg.

Table 3. Univariate and multivariate analysis of overall survival in patients with PAP (N=484).

| **Characteristics** | **Univariate analysis** | | | **Multivariable analysis** | | |
| --- | --- | --- | --- | --- | --- | --- |
|  | **HR** | **95%CI** | **p-value** | **HR** | **95%CI** | **p-value** |
| **Age** |  |  |  |  |  |  |
| ＜60 | 1 |  |  | 1 |  |  |
| ≥60 | 1..903 | 1.211-2.990 | **0.005** | 1.837 | 1.150-2.934 | **0.011** |
| **Gender** |  |  |  |  |  |  |
| Male | 1 |  |  | 1 |  |  |
| Female | 0.783 | 0.571-1.074 | 0.129 | 0.804 | 0.580-1.115 | 0.191 |
| **Race** |  |  | 0.908 |  |  | 0.722 |
| White | 1 |  |  | 1 |  |  |
| Black | 1.129 | 0.649-1.962 | 0.668 | 1.295 | 0.714-2.221 | 0.426 |
| Others | 0.99 | 0.587-1.667 | 0.969 | 0.992 | 0.572-1.721 | 0.976 |
| **T stage** |  |  |  |  |  |  |
| T1 | 1 |  |  | 1 |  |  |
| T2 | 1.74 | 1.268-2.388 | 0.001 | 1.192 | 0.677-2.096 | 0.543 |
| **Grade** |  |  | 0.171 |  |  | 0.182 |
| Well or moderatelydifferentiated | 1 |  |  | 1 |  |  |
| Poorly or undifferentiated | 1.489 | 0.823-2.694 | 0.189 | 1.382 | 0.735-2.599 | 0.315 |
| Unknown | 1.533 | 0.864-2.720 | 0.144 | 1.627 | 0.908-2.918 | 0.102 |
| **Tumor size** |  |  |  |  |  | 0.214 |
| ≤3 | 1 |  |  | 1 |  |  |
| ＞3 | 1.863 | 1.356-2.559 | **＜0.001** | 1.424 | 0.816-2.484 | 0.214 |
| **Radiotherapy** |  |  |  |  |  |  |
| No | 1 |  |  | 1 |  |  |
| Yes | 1.926 | 0.613-6.047 | 0.262 | 2.501 | 0.771-8.113 | 0.127 |
| **Chemotherapy** |  |  |  |  |  |  |
| No/Unknown | 1 |  |  | 1 |  |  |
| Yes | 1.747 | 1.122-2.721 | **0.014** | 1.532 | 0.938-2.504 | 0.089 |
| **Surgery** |  |  | 0.371 |  |  | 0.211 |
| Wed | 1 |  |  | 1 |  |  |
| Seg | 1.066 | 0.442-2.573 | 0.887 | 1.215 | 0.496-2.975 | 0.67 |
| Lob^†^ | 0.771 | 0.513-1.159 | 0.211 | 0.735 | 0.481-1.123 | 0.154 |
| Lob^‡^ | 0.723 | 0.318-1.648 | 0.441 | 0.605 | 0.263-1.393 | 0.237 |

Abbreviation: PAP: papillary predominant adenocarcinoma; HR, hazard ratio; CI, confidence interval; Wed: wedge resection; Seg: segmentectomy; Lob: lobectomy with mediastinal lymph node dissection; †: Lob was compared to Wed; ‡: Lob was compared to Seg.

Supplemental table 4. Univariate and multivariate analysis of lung cancer specific survival in patients with PAP (N = 484).

| **Characteristics** | **Univariate analysis** | | | **Multivariate analysis** | | |
| --- | --- | --- | --- | --- | --- | --- |
|  | **HR** | **95% CI** | ***p*-value** | **HR** | **95% CI** | ***p*-value** |
| **Age** |  |  |  |  |  |  |
| ＜60 | 1 |  |  | 1 |  |  |
| ≥60 | 1.643 | 0.946-2.854 | 0.078 | 1.561 | 0.879-2.774 | 0.129 |
| **Gender** |  |  |  |  |  |  |
| Male | 1 |  |  | 1 |  |  |
| Female | 0.882 | 0.590-1.319 | 0.541 | 0.881 | 0.578-1.343 | 0.557 |
| **Race** |  |  | 0.652 |  |  | 0.866 |
| White | 1 |  |  | 1 |  |  |
| Black | 0.911 | 0.420-1.978 | 0.814 | 0.95 | 0.429-2.107 | 0.9 |
| Others | 1.296 | 0.719-2.337 | 0.389 | 1.176 | 0.623-2.221 | 0.617 |
| **T stage** |  |  |  |  |  |  |
| T1 | 1 |  |  | 1 |  |  |
| T2 | 2.374 | 1.568-3.594 | **＜0.001** | 1.819 | 0.947-3.494 | 0.072 |
| **Grade** |  |  | **0.004** |  |  | **0.009** |
| Well or moderately differentiated | 1 |  |  | 1 |  |  |
| Poorly or undifferentiated | 2.313 | 1.226-4.365 | **0.01** | 1.897 | 0.955-3.768 | 0.068 |
| Unknown | 2.168 | 1.147-4.096 | **0.017** | 2.454 | 1.275-4.725 | **0.007** |
| **Tumor size** |  |  |  |  |  |  |
| ≤3 | 1 |  |  | 1 |  |  |
| ＞3 | 2.134 | 1.426-3.193 | **＜0.001** | 1.113 | 0.599-2.066 | 0.736 |
| **Radiotherapy** |  |  |  |  |  |  |
| No | 1 |  |  | 1 |  |  |
| Yes | 0.97 | 0.135-6.962 | 0.976 | 1.429 | 0.193-10.574 | 0.727 |
| **Chemotherapy** |  |  |  |  |  |  |
| No/Unknown | 1 |  |  | 1 |  |  |
| Yes | 2.349 | 1.406-3.925 | **0.001** | 2.049 | 1.153-3.643 | **0.015** |
| **Surgery** |  |  | 0.557 |  |  | 0.233 |
| Wed | 1 |  |  | 1 |  |  |
| Seg | 1.109 | 0.374-3.284 | 0.852 | 1.273 | 0.423-3.835 | 0.668 |
| Lob^†^ | 0.786 | 0.469-1.318 | 0.361 | 0.688 | 0.402-1.180 | 0.174 |
| Lob^‡^ | 0.709 | 0.258-1.944 | 0.504 | 0.541 | 0.194-1.504 | 0.239 |

Abbreviation: PAP: papillary predominant adenocarcinoma; HR, hazard ratio; CI, confidence interval; Wed: wedge resection; Seg: segmentectomy; Lob: lobectomy with mediastinal lymph node dissection; †: Lob was compared to Wed; ‡: Lob was compared to Seg.
